# Complete genomic analysis of *Escherichia* phage Mangalyan infecting *Escherichia fergusonii*

**DOI:** 10.1128/mra.00963-23

**Published:** 2023-12-13

**Authors:** Prasanth Manohar, Ry Young

**Affiliations:** 1 Department of Biochemistry and Biophysics, Center for Phage Technology, Texas A&M AgriLife Research, Texas A&M University, College Station, Texas, USA; Queens College, Queens, New York, USA

**Keywords:** bacteriophage genetics, bacteriophages

## Abstract

*Escherichia fergusonii* is a rarely isolated opportunistic pathogen in animals and humans. Here, we present the annotated genome sequence of *Escherichia* phage Mangalyan, a T4-like bacteriophage infecting *E. fergusonii* isolated from chickens. Phage Mangalyan has a genome length of 140,513 bp and belongs to the *Vequintavirinae* family.

## ANNOUNCEMENT

*Escherichia fergusonii* is one of the emerging human pathogens and is known to transmit via the food chain (1). It causes infections in humans such as bacteremia, diarrhea, wound infections, and urinary tract infections (2). *E. fergusonii* is common in chickens. The *E. fergusonii* strain RY44 (https://www.ncbi.nlm.nih.gov/nuccore/NZ_JASJUP000000000.1) used in this study was isolated from chicken ceca samples collected at the Poultry Science facility at Texas A&M University (30.62°N, 96.33°W) in College Station, TX, USA.

For the isolation of bacteriophages against *E. fergusonii*, chicken fecal samples isolated from the same facility were filtered through a 0.22-micron pore-size filter (MilliporeSigma) to create a virus sample of 100 mL and RY44 for 24 h in lysogeny broth at 37°C with aeration. Clear plaques were obtained with a soft agar overlay method (3). Phage morphology was determined using negative-stain transmission electron microscopy performed at the Texas A&M University Microscopy and Imaging Center, as described in reference (4). Phage DNA was isolated from the single plaque-purified phages using a phenol-chloroform method as described in reference (5). Illumina sequencing libraries were prepared using the tagmentation-based and PCR-based Illumina DNA Prep kit and custom IDT 10 bp unique dual indices with a target insert size of 320 bp. Sequencing was performed on an Illumina NovaSeq 6000 sequencer in one or more multiplexed shared-flow-cell runs (one run), producing 151 bp paired-end reads. Demultiplexing, quality control, and adapter trimming were performed with bcl-convert1 (v4.1.5), resulting in 1,329,248 reads for a total coverage of 1,428×.

Genome assembly and annotation were performed using Galaxy (6) and Web Apollo (7), hosted by the Center for Phage Technology (8). The raw reads were quality controlled using FastQC (Galaxy v0.72 + galaxy1) (https://www.bioinformatics.babraham.ac.uk/projects/fastqc/) and trimmed with the trimmomatic (Galaxy v0.38.0) (9) toolkit. The reads were assembled using SPAdes v3.12.0 + galaxy1 (10). Contig completion was confirmed using the PCR primers (5′-CTTTCGTCGGTGGTCCACAT-3′ and 5′-CTGCTACCTGCACAACTGGT-3′) and Sanger sequencing reads (11). Galaxy’s Structural Annotation PAP Workflow v2023.01 was used to predict potential genes, using GLIMMER v3 (12), MetaGeneAnnotator (13), and Sixpack, and ARAGORN v2.36 (14) and tRNAScan-SE v2.0 for tRNA prediction. The Functional Annotation PAP Workflow v2023.01 was used to predict the gene functions from three databases: Canonical Phages, Swiss-Prot, and Nonredundant Phages. InterProScan v5.48 (15), BLAST v2.9.0 (16), TMHMM v2.0 (17), HHpred, LipoP v1.0 (18), and SignalP v5.0 were used to resolve conflicting evidence. All tools were run with default settings.

**Fig 1:**
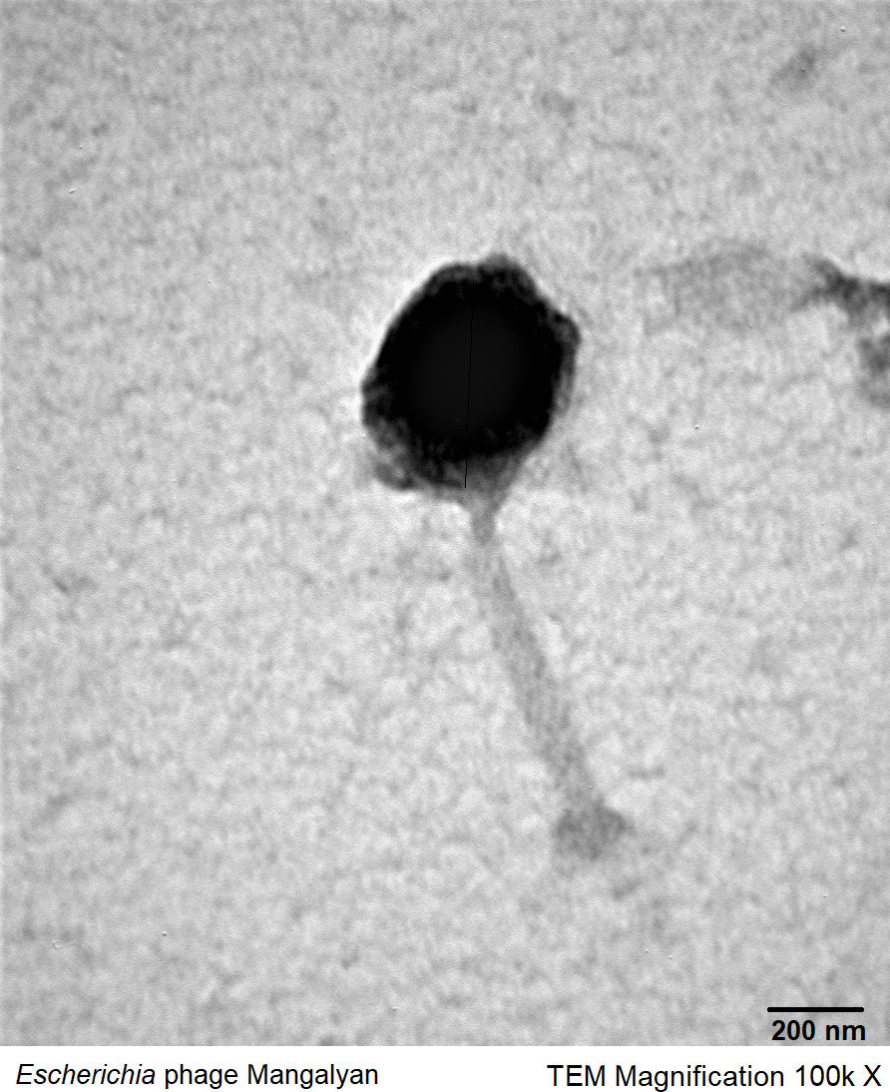
TEM of phage Mangalyan infecting *Escherichia fergusonii*. TEM magnification of 100,000×.

Phage Mangalyan has a genome of 140,513 bp, a GC content of 43.57%, and is assembled at 657× coverage. It has 239 coding sequence (CDS), of which 83 have predicted functions, and 7 tRNA genes. Mangalyan was determined to have T4-type morphology using transmission electron micrograph (TEM) analysis (Fig. 1). Based on blastn results, Mangalyan showed >98% and 96% similarities and 94% and 96% query coverage to *Escherichia* phage CarlMeissner (MZ501054) and *Escherichia* phage naswa (MN850595), respectively. Therefore, it belongs to the *Vequintavirinae* family, a subfamily of Vequintavirus.

## Data Availability

The genome sequence of phage Mangalyan has been submitted to GenBank under the accession number OR449013. The associated BioProject, SRA, and BioSample accession numbers are PRJNA988702, SRR25175890, and SAMN36026652, respectively.
